# The Open Spectral Database: an open platform for sharing and searching spectral data

**DOI:** 10.1186/s13321-016-0170-2

**Published:** 2016-10-14

**Authors:** Stuart J. Chalk

**Affiliations:** Department of Chemistry, University of North Florida, Jacksonville, FL 32224 USA

**Keywords:** Spectral data, REST API, Open science, Open data, JCAMP-DX, XML, Scientific data model

## Abstract

**Background:**

A number of websites make available spectral data for download (typically as JCAMP-DX text files) and one (ChemSpider) that also allows users to contribute spectral files. As a result, searching and retrieving such spectral data can be time consuming, and difficult to reuse if the data is compressed in the JCAMP-DX file. What is needed is a single resource that allows submission of JCAMP-DX files, export of the raw data in multiple formats, searching based on multiple chemical identifiers, and is open in terms of license and access. To address these issues a new online resource called the Open Spectral Database (OSDB) http://osdb.info/ has been developed and is now available. Built using open source tools, using open code (hosted on GitHub), providing open data, and open to community input about design and functionality, the OSDB is available for anyone to submit spectral data, making it searchable and available to the scientific community. This paper details the concept and coding, internal architecture, export formats, Representational State Transfer (REST) Application Programming Interface and options for submission of data.

**Results:**

The OSDB website went live in November 2015. Concurrently, the GitHub repository was made available at https://github.com/stuchalk/OSDB/, and is open for collaborators to join the project, submit issues, and contribute code.

**Conclusion:**

The combination of a scripting environment (PHPStorm), a PHP Framework (CakePHP), a relational database (MySQL) and a code repository (GitHub) provides all the capabilities to easily develop REST based websites for ingestion, curation and exposure of open chemical data to the community at all levels. It is hoped this software stack (or equivalent ones in other scripting languages) will be leveraged to make more chemical data available for both humans and computers.

## Background

Tools to make research data freely available are vitally important to the open science movement. Such tools must play well with both humans and computers because of the importance of data import/export into other systems for analysis, verification, and data mining. One important data type in this area is instrumental spectra, used for identification and analysis in a variety of different application areas. Many websites (e.g. NIST Webbook [1], ChemSpider [2], University of the West Indies—Chemistry [3]) contain spectral files available in the current de-facto data standard, Joint Committee on Atomic and Molecular Physical Data—Data Exchange format (JCAMP-DX) [4–7] and this format can be exported from the majority of instrument software available today. However, the usefulness of spectral data in JCAMP-DX format is somewhat limited due to the specification being over 30 years old, and if saved using compression, difficult to transfer to other software. Providing a mechanism to allow conversion of legacy data in JCAMP-DX format is an important activity in-of-itself, as the community needs spectral data for comparison/standardization in many different applications.

In order to leverage data in JCAMP-DX and make it more easily available and searchable, a website has been developed [8] that allows (1) upload of JCAMP-DX files, (2) extraction and conversion of the data and metadata to an extensible markup language (XML) pseudo JCAMP-DX equivalent, and (3) encoding of the data in JavaScript Object Notation for Linked Data (JSON-LD) [9] using a generic scientific data model (SDM) [10]. Each of these three formats is available for download.

The website has been developed using open-source software (as far as possible), using open standards, and is openly being made available using the GitHub code repository. The website is built in the Representational State Transfer (REST) style [11] and has a documented Application Programming Interface (API) [12] for computer based discovery and export.

## Implementation

The foundation of the OSDB website is the common Apache [13], MySQL [14], and PHP [15] software stack that can be installed on any computer system as: LAMP (for Linux), WAMP (for Windows) and MAMP [for OSX (Mac)]. Coding was done using the PHPStorm [16] Integrated Development Environment (IDE) (free for faculty and students) and scripts are written in PHP implementing the CakePHP object oriented framework [17]. Because of the use of this standard open-source software developers can either; deploy on their own physical server, publish using one of a number of online hosting sites, or use a virtual machine, for creation of data websites.

### Website functionality

**Fig. 1:**
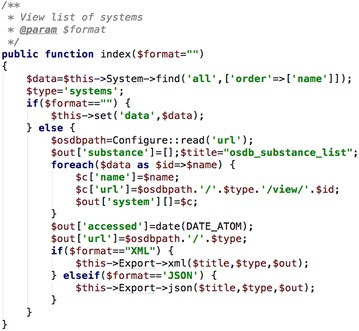
CakePHP controller code to retrieve chemical system data and pass it to the view file

As the goal of the project was to develop a site that offered a standardized REST style API, the PHP framework CakePHP was used to develop the code. CakePHP standardizes the development of PHP scripts by use of the model–view–controller (MVC) [18] model, implemented using Object Oriented Programming (OOP) [19]. The MVC paradigm separates code into logical sections; the model—to access a database table, the view—presentation of data as a web page, and the controller—the logic that coordinates the processing of a webpage request into a Hypertext Markup Language (HTML) document. As an example, if the user goes to the systems index page [20], then the following code is executed (Fig. 1).

**Fig. 2:**
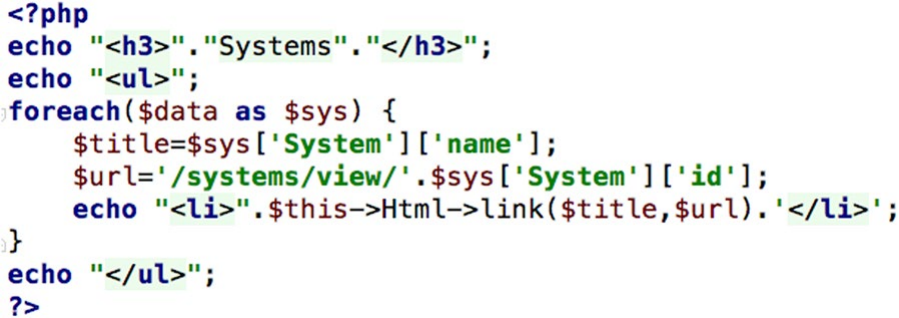
PHP snippet used to display and index of chemical systems

The function ‘index’ in the SystemsController.php file executes a set of PHP commands to present a list of current chemical systems in the database, and is one of a number of methods of the SystemsController class. This is executed by default where is no action after “/systems” in the URL. In the first line the call to $this->System->find accesses the System ‘model’ (that accesses the ‘systems’ database table) and executes the find ‘method’ to retrieve all the systems, and place the data in the $data variable. The $this->set command assigns the data returned to a variable called ‘data’ that will be available to the PHP code in the view file. For “/systems”, once the code in the controller has finished, CakePHP knows to then return the ‘index.ctp’ file (CakePHP template file) to the browser—shown in Fig. 2.

Note that the function in Fig. 1 has a variable ($format) in the function arguments and when no value is passed the default of an empty string is set. When $format is tested as being equal to an empty string the $this->set command completes and the HTML file ‘index.ctp’ is rendered. However, if the URL “/systems/index/XML” is accessed, the same call is made except the data is not sent to the view and is instead is converted to an array and reformatted as XML ($this->Export->xml()) and passed to the browser. Note that the action ‘index’ must be included in the URL so that CakePHP does not try and run the action ‘XML’ (i.e. “/systems/XML” will cause an error).

**Fig. 3:**
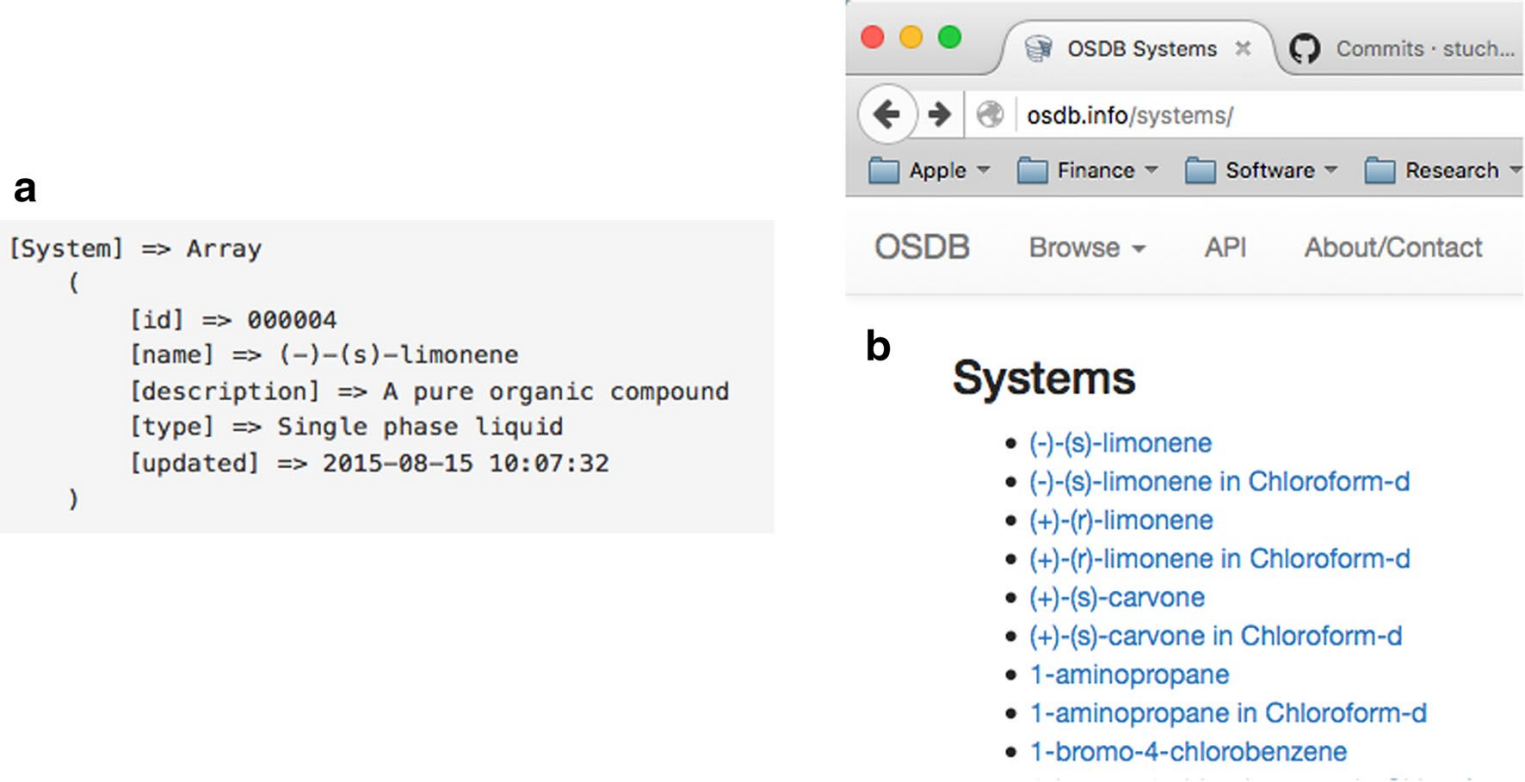
System data in **a** PHP array, **b** a web page

The view file takes the list of all the systems in $data (view variable), iterates through each one ($data is a PHP array—see Fig. 3a) and prints out a HTML link on the webpage as an unordered list “<ul>” and shown in Fig. 3b.

### Spectral file format

The Joint Committee on Atomic and Molecular Physical Data (JCAMP) published the specification for the Data eXchange format (DX) for spectral data for UV/Vis and IR [6], MS [7], IMS [21], NMR [5], ESR [22], and CD [23] data. JCAMP-DX files are ASCII text files populated with LABELLED-DATA-RECORDs or LDRs. These are defined to allow reporting of spectral metadata and raw/processed instrument data. The instrument data is reported as XY pairs, nominally in tabular format where one line contains a starting X value and a number of equally spaced Y values, the X value of which can be calculated using the LDR DELTAX. In addition, because the format was developed when disk space was at a premium, the data can be reported in a number of compressed formats, referred to as ASCII Squeezed Difference Form (ASDF), that use letters and symbols to encode data in more compact formats (Table 1).

**Table 1:** Pseudo-digits for ASDF

1. ASCII digits	0	1	2	3	4	5	6	7	8	9
2. Positive SQZ digits	@	A	B	C	D	E	F	G	H	I
3. Negative SQZ digits		a	b	c	d	e	f	g	h	i
4. Positive DIF digits	%	J	j	L	M	N	O	P	Q	R
5. Negative DIF digits		j	l	l	m	n	o	p	q	r
6. Positive DUP digits		S	T	U	V	V	W	X	Y	Z

 Table 2 shows data in normal fixed format and equivalent storage in four compression formats.

**Table 2:** Example of ASDF Formats (only Y data points shown)

FIX form: (22 chars)	1	2	3	3	2	1	0	−1	−2	−3
PAC form: (19 chars)	1 + 2 + 3 + 3 + 2 + 1 + 0 − 1 − 2 − 3									
Or:	1 2 3 3 2 1 0 − 1 − 2 − 3									
SQZ form: (10 chars)	1BCCBA@abc									
DIF form: (10 chars)	1JJ%jjjjjj									
DIFDUP form: (7 chars)	1JT%jX									

When a JCAMP file is uploaded to the OSDB website, a record is added to the database that contains the file metadata. A unique id is generated and the file is saved with the id as its filename (available at id.jdx). The file is then read into a variable in PHP and processed line by line using a JCAMP plugin written in our laboratory. The plugin processes the file using the following seven steps:

- Clean—remove non-ACSII characters extra spaces at the start/end of lines
- Uncomment—remove (and save) comments (indicated by $$)
- Get LDRs—detect LDRs in the file
- Validate—check the LDRs to identify if the file is valid JCAMP-DX
- Standardize—standardize data in certain LDR fields
- Decompress—expand data in any of the ASDF formats and calculates respective X values

**Fig. 4:**
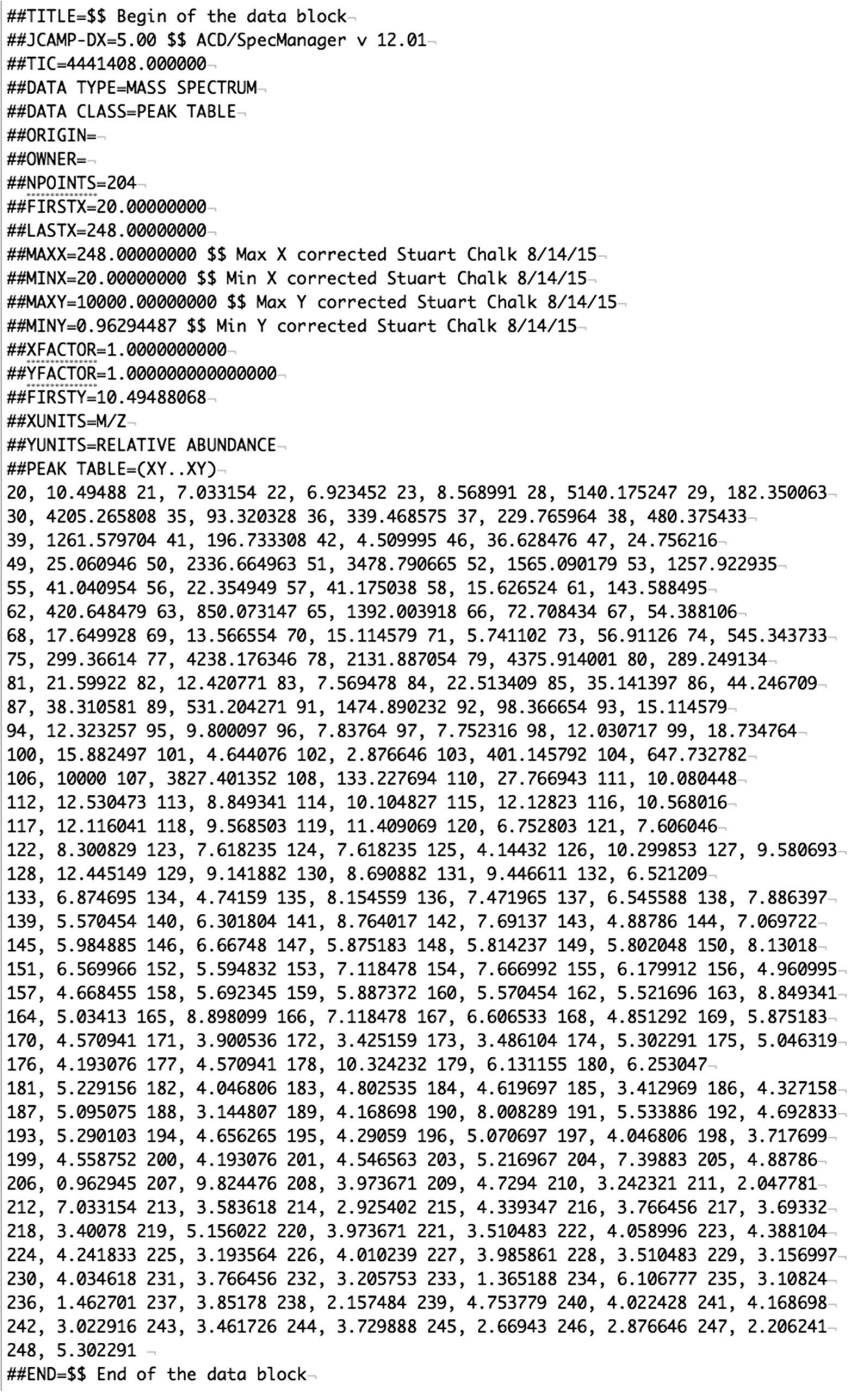
JCAMP-DX file

**Fig. 5:**
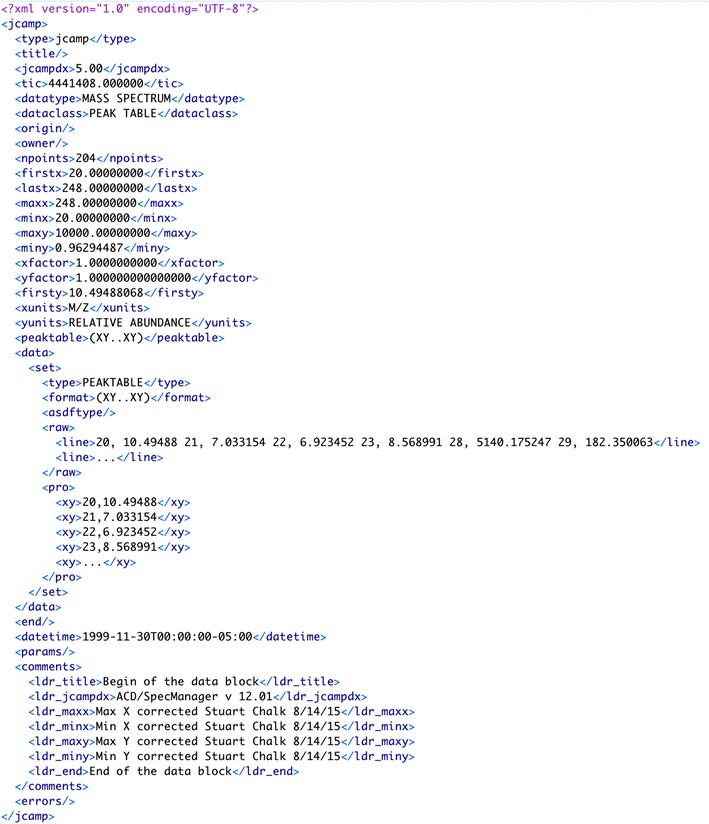
JCAMP-DX file in XML format

The metadata, data, comments and any processing errors are stored in an array in PHP and then converted to XML and saved. Figures 4 and 5 show a comparison of the original JCAMP file (e.g. “../spectra/view/000000115/JCAMP”) and JCAMP saved in XML (e.g. “../spectra/view/000000115/XML”).

Spectral data in the JCAMP file is stored both in its <raw> state along with the <pro> (cessed) expanded format. Any discrepancies between the data in the original JCAMP file and the process data are annotated in the errors element, with details of the issues.

### Data management

**Fig. 6:**
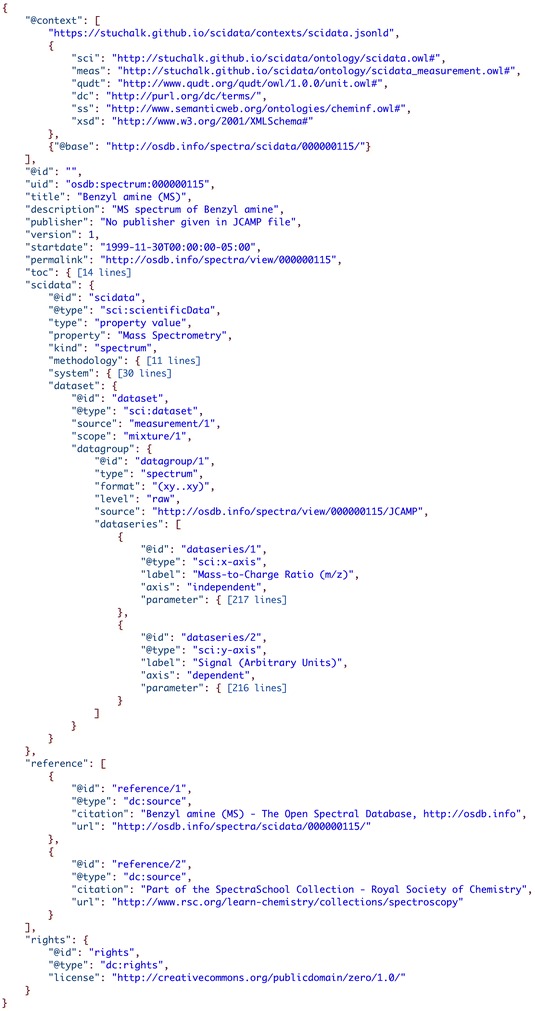
Spectra data in the SciData scientific data model format

The spectral data is also saved in a MySQL database designed around the SDM outlined in the recent paper [24]. The SDM is a generic framework of organizing the data and metadata obtained in scientific experiments. In a nutshell, the SDM describes data and its metadata containers that can be used to aggregate scientific data and metadata and relate them together. It can be implemented in any database, XML Markup Language, or text format, for example in JavaScript Object Notation for Linked Data, JSON-LD [9]. If implemented in JSON-LD the information can be semantically represented using a set of SDM JSON-LD context files (see Fig. 6). More information and examples of the SDM format can be found on the projects website [10].

### Graphical user interface

A common problem currently with website design is building a graphical user interface (GUI) that works equally well on computers, tablets, and phones. Thankfully, the developers of Twitter have built a free CSS/JavaScript solution to this problem called Bootstrap [25]. Bootstrap uses predefined Cascading Style Sheet (CSS) [26] classes that are designed to work with HTML 5 [27] and plugins for the popular jQuery [28] JavaScript library. As a result, the basic Bootstrap implementation produces a clean, browser natural, device neutral GUI that minimizes the development of the site interface.

## Results

The website allows users to access the data in the system via endpoints (MVC controller) for:

- Systems [20]
- Spectra [29]
- Compounds [30]
- (Analytical) Techniques [31]
- Collections [32]

**Fig. 7:**
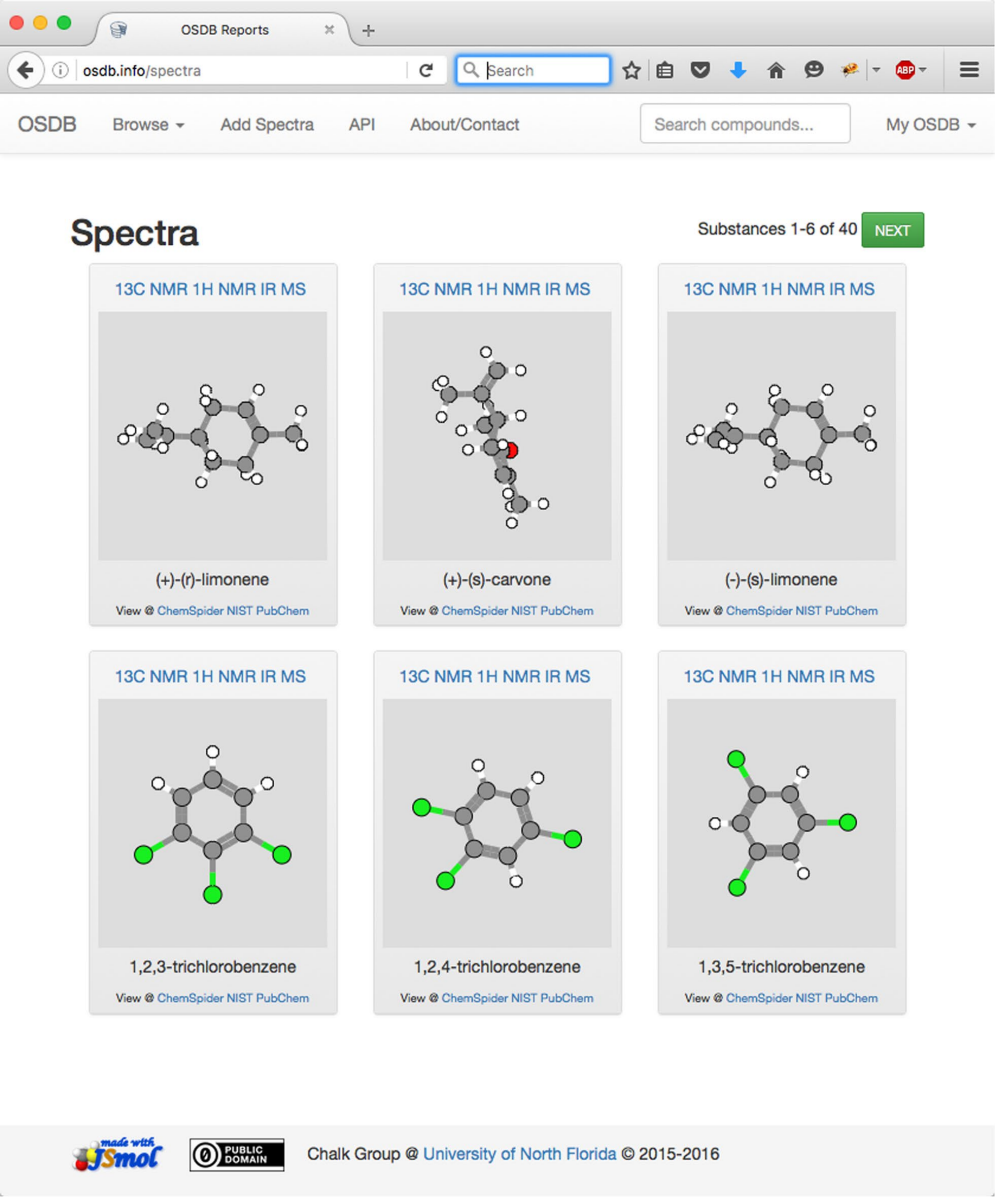
OSDB spectral index page

The default pages accessed via “/<controllername>” URLs all provide an index of available resources of that type. For instance, Fig. 7 shows the spectra index, with spectra organized by compound, with a JSmol [33] view of the structure, a link to the compound page, and external links to more data about the compound.

In addition to the index view for spectra, users can search of a specific compound using the search box at the top of the page. The search is performed over the ‘identifier’ database table which is populated from the PubChem PUGREST interface [34] and contains names, SMILES, PubChem CIDs, InChI strings and InChIKeys.

**Fig. 8:**
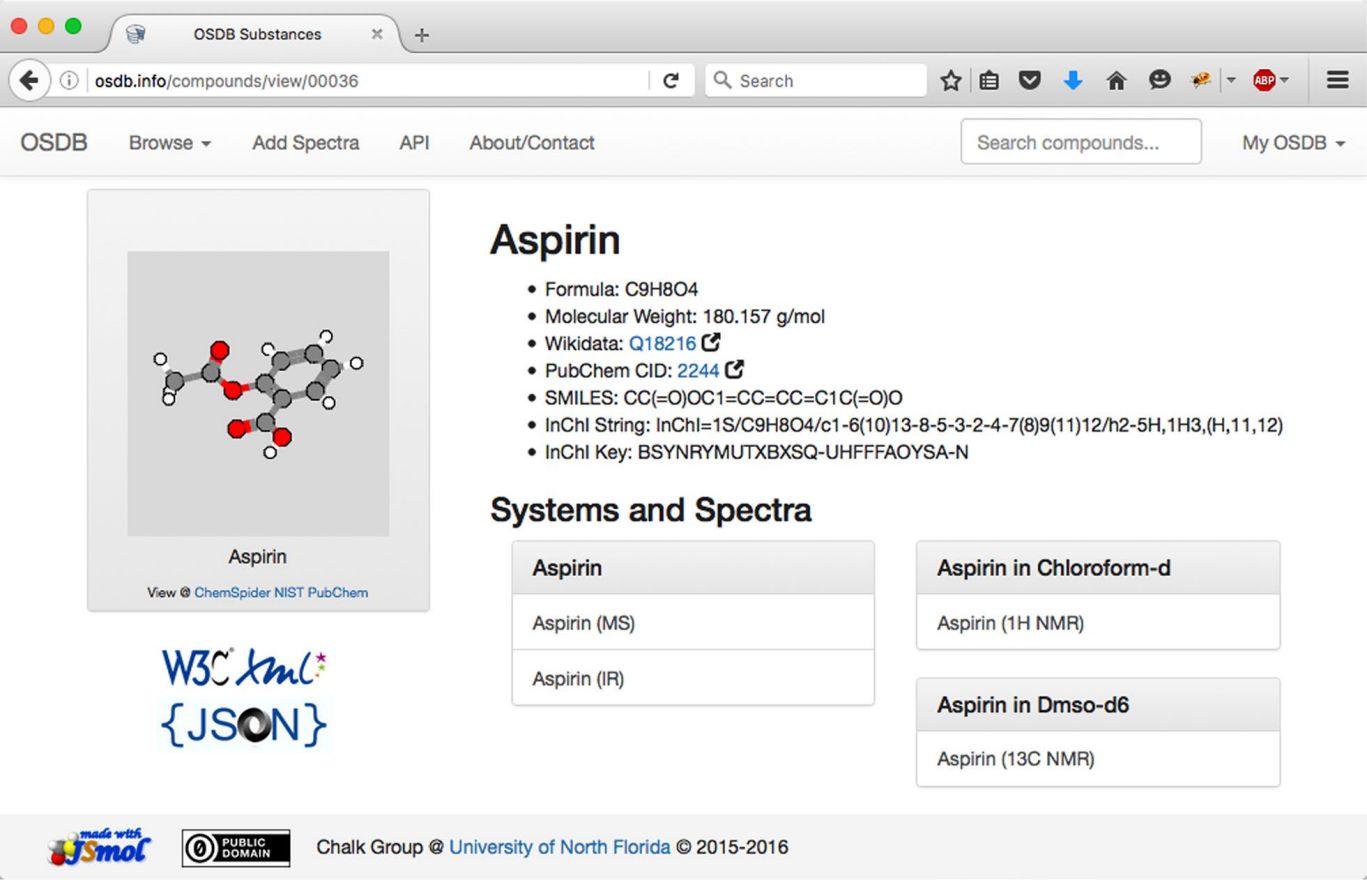
OSDB view of data for Aspirin

Clicking on the name of a compound on the compound index page [30] brings up a summary page of a compound with the JSmol molecular view, metadata, and links to the spectra for that compound. Figure 8 shows the page for aspirin. Note the external links to view the chemical on PubChem [35] and Wikidata [36] a free linked database that underpins a number of websites including Wikipedia.

**Fig. 9:**
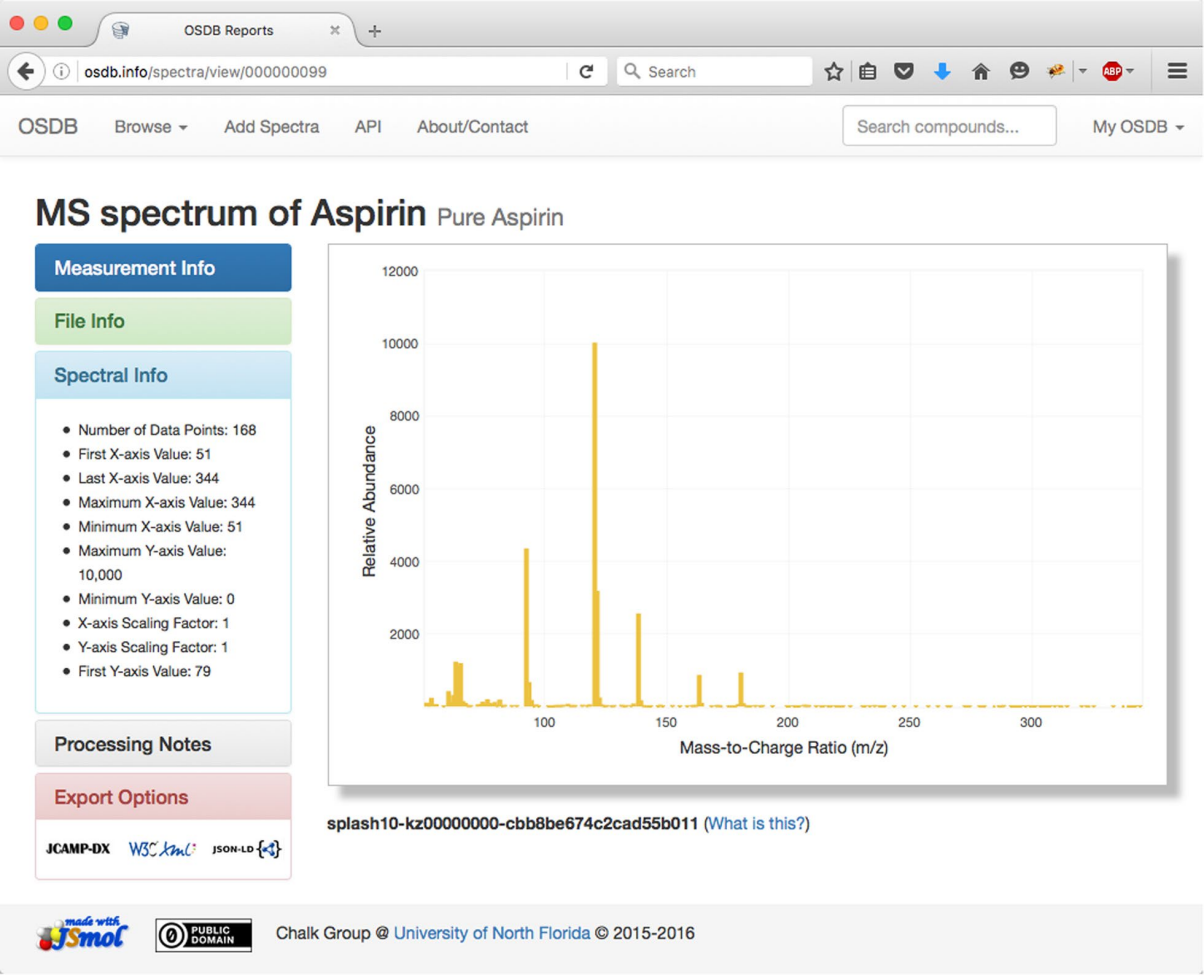
MS spectrum of aspirin

Clicking on a spectral link (under Systems and Spectra) brings up the view of the spectrum (see Fig. 9 for the MS spectrum of Aspirin). Metadata about the spectrum is available on the left side by clicking each of the four buttons. The spectrum can also be downloaded in JCAMP, XML, and SciData (JSON-LD) formats by clicking the respective icon.

**Fig. 10:**
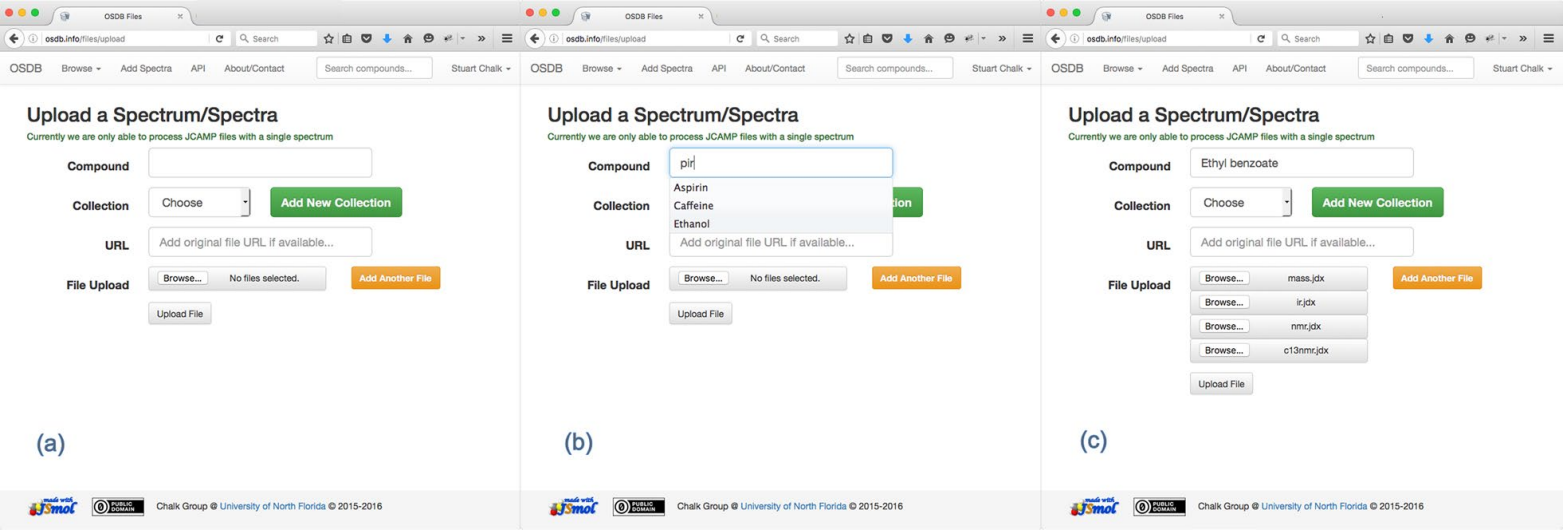
Uploading a file to the OSDB.** a** Upload page,** b** compound search suggestions, and** c** adding multiple files for upload

To contribute a spectrum to the OSDB, users first signup for an account under “My OSDB” (top right) and then click on “Add Spectra” on the top menu bar. The form for upload of JCAMP files (Fig. 10a) can be used to upload a file from a local drive or a web address. When entering the compound name the form searches and displays (Fig. 10b) the existing compounds in the system and clicking on one of the names found selects that compound. If the user is uploading local files they can add as many as they like by clicking the “Add another file” button (Fig. 10c).

**Fig. 11:**
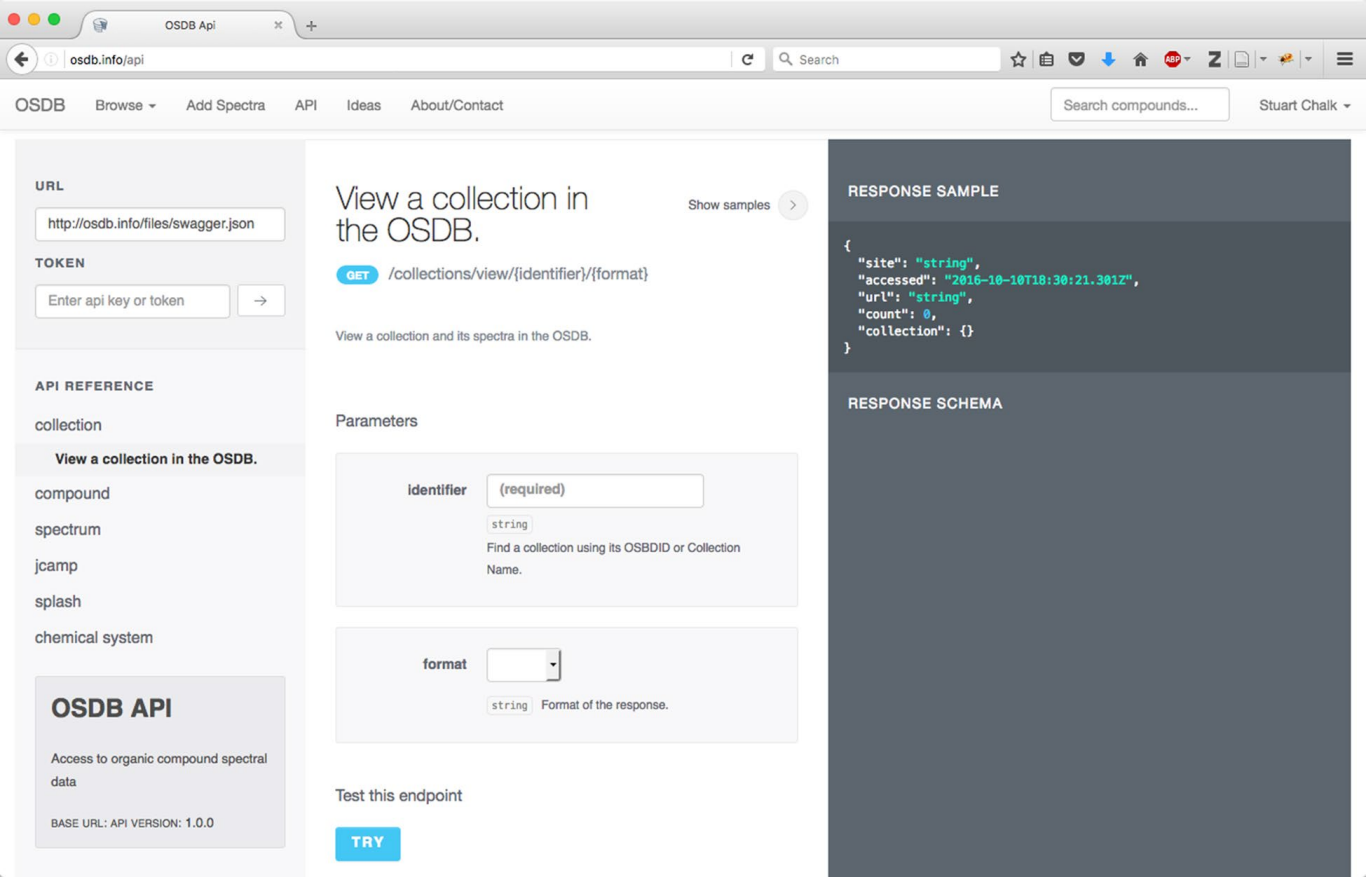
The OSDB REST API specification

For computer access to all of the above functionality (except file upload) a REST API is available and described here [37] (Fig. 11) built using the widely popular Swagger API framework [38]. As an example for spectral data the API allows access to the files via a number of formats—OSDB ID, Splash [39], and compound name and technique code (comp|tech). It is also possible to access just the plot of the spectrum which is useful for embedding the data into another website.

### Features

The basic REST website provides a mechanism to add data to the repository, access it in a standardize way and download the data in multiple formats. However, the key to making the data truly accessible is by integration with other platforms and expanded search capabilities. These features have been added to the OSDB website through the following additions.

#### PubChem lookup for chemical metadata

When a new compound is entered on the spectra upload page and new spectrum uploaded the system does a check for the compound in the current system and if not found searches PubChem using the Power User Gateway REST API [34]. PubChem allows extensive searching of the data and metadata the system holds via the API, which has a myriad of options and has the generalized URL.

“http://pubchem.ncbi.nlm.nih.gov/rest/pug/

<inputspecification>/<operation specification>/[<output specification>][?<operation_options>]”

**Fig. 12:**
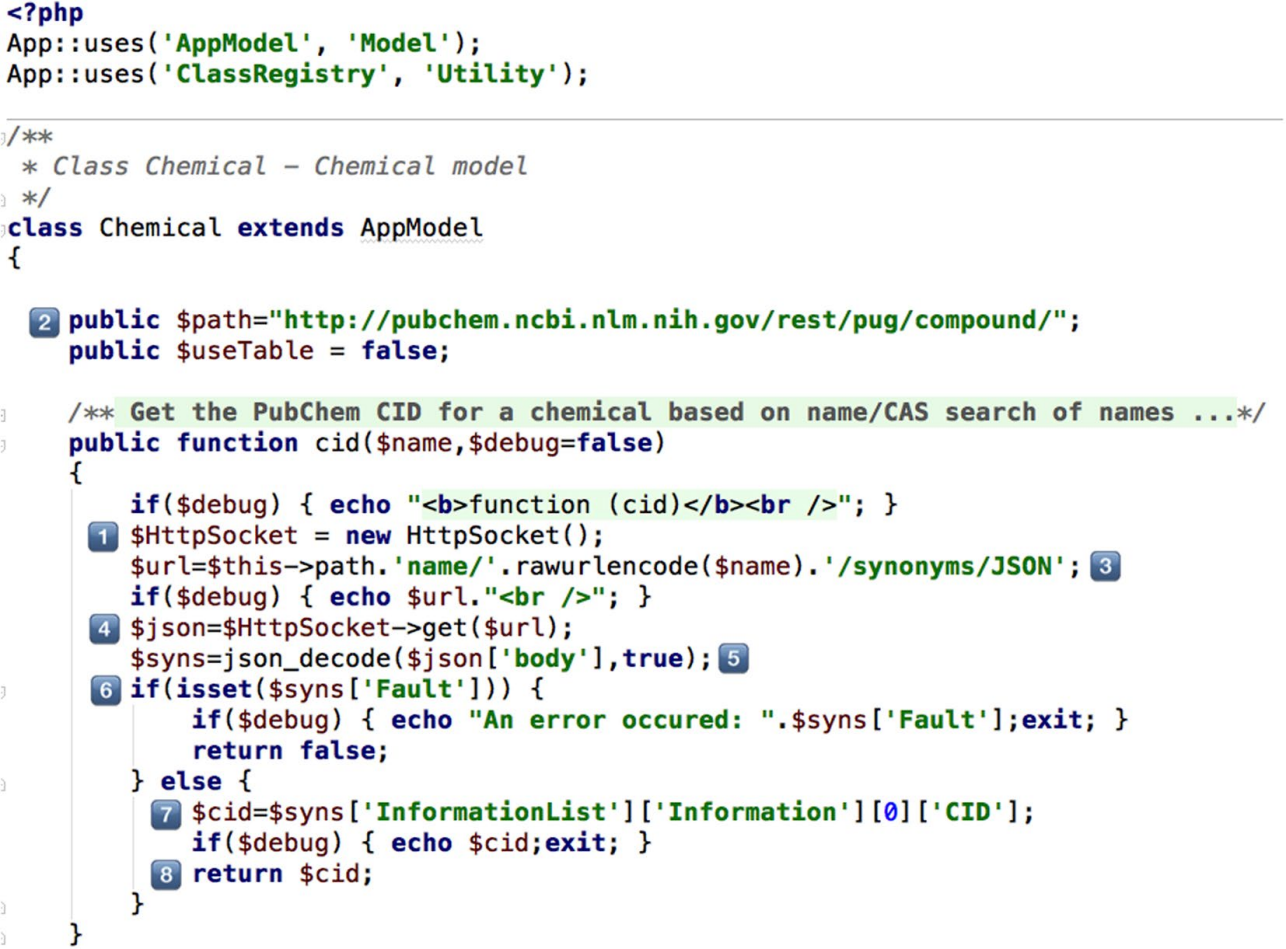
Function to search the PubChem REST API for chemical names and CAS #’s

As an example of using this API to gather data about compounds, users submit the compound name along with the spectral data to the OSDB. Figure 12 shows a PHP function written to allow the system to retrieve the PubChem CID for the compound entered, which is subsequently used to retrieve the identifier data mentioned earlier.

The function cid has two arguments $name, and $debug (used to check that the code is working correctly). First, access to the CakePHP HttpSocket is established [4], and URL constructed from the base PubChem API address [2], the compound name ($name), and ‘/synonym/JSON’ [8]. The URL is requested (equivalent to a web browser) [40] and the resulting JSON data converted to a PHP array $syns [3]. The code then checks for errors in the response [3] and retrieves the CID for the compound [5]. The value of the CID is then returned to the calling function. Other functions in the Chemical class return all the synonyms for a compound, and property data for a compound.

#### Retrieve Wikidata ID

**Fig. 13:**
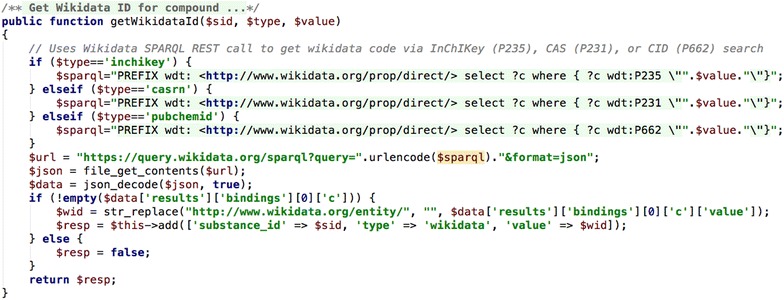
CakePHP function to search and retrieve the Wikidata ID for a compound

Similar to the PubChem example, a function was written to search the Wikidata website [36], this time using a SPARQL query [41] encoded in a URL (Fig. 13). Three separate searches are coded to retrieve the Wikidata ID via InChIKey, SMILES, or PubChem CID. If the script calling this function tries all three approaches and does not get an ID, it assumes that the compound is not in the Wikidata database.

#### Generate the Splash for a spectrum

**Fig. 14:**
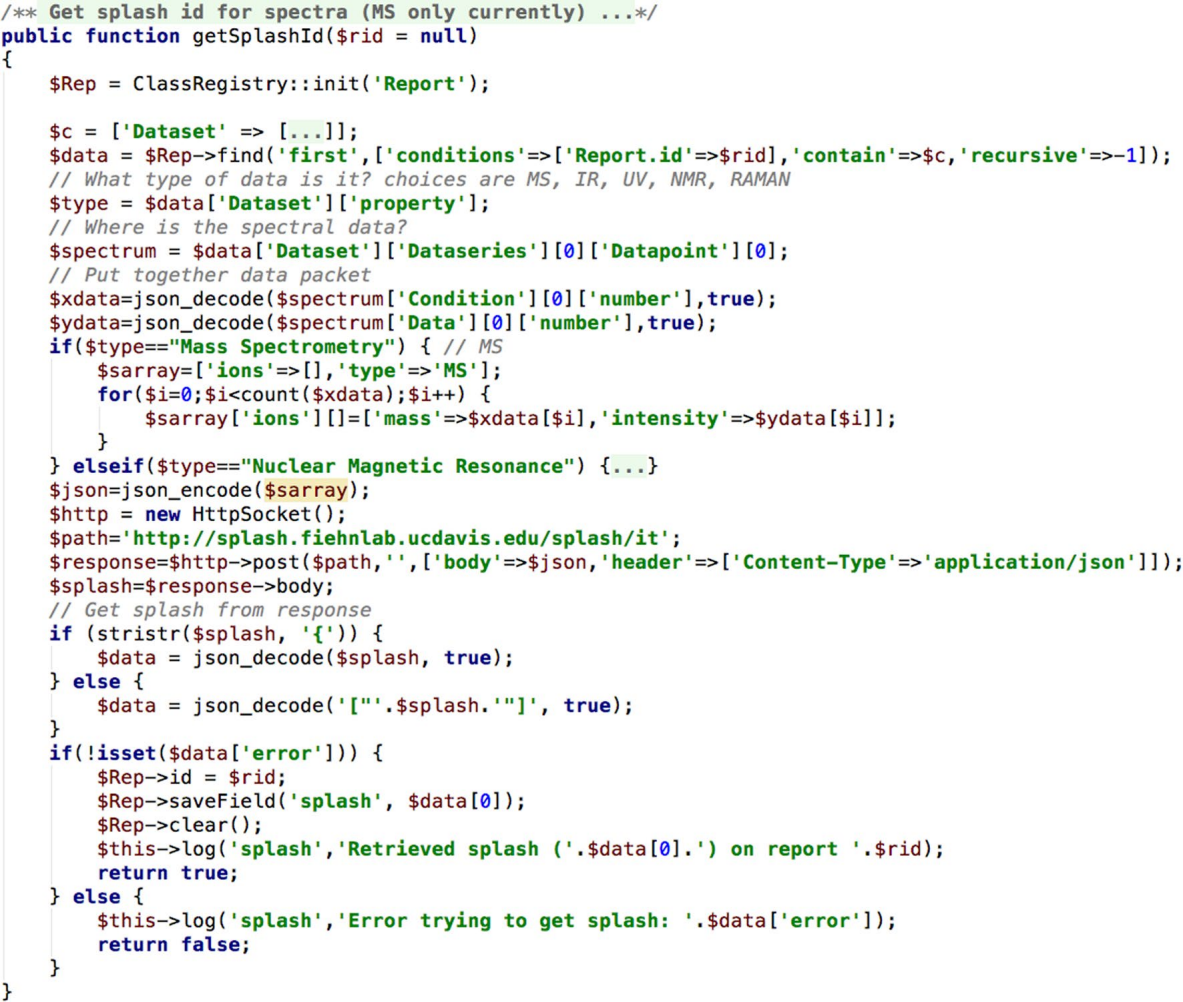
CakePHP code to generate a Splash identifier

A recent addition to the identifier scene is the ‘Spectral Hash’ or Splash [39]. This identifier evolved out of work started at the 2015 Metabolomics Hackathon [42] where participants became enthusiastic about unique spectral identifiers similar to the InChIKey. In order to generate a Splash for a spectrum the spectral data is encoded in a JSON object and then sent to the Splash website. The code in Fig. 14 does just that.

## Discussion

The OSDB website, as outlined above, provides access to spectral data and its metadata in a standardize way. However, it is important to point out that what can be done with the data is up to the user. This applies to the OSDB website as well as after spectra have been downloaded. For instance, the website does not currently allow for searching the raw data/metadata across all spectra (all of it is in the database but can only be found searching for a complete spectrum).

In order to make this site truly useful the code and data of the project should be made openly available. In this way the user is not limited to the functionality that the original developers envisioned but can develop their own functions/features, enhance the integration of the site, and output the data in new formats for new web or mobile applications. In addition, the openness of the project means it can be used in education as a tool to develop the next generation of cheminformaticians—potentially building their own website from the source code as a course project.

For all these reasons (and many more) the project is available as a free download on GitHub [40]. GitHub is a hosting service for the well-respected Git source code repository system [43]. Git allows multiple developers to write code for one project and centrally coordinate version control, patching, extension and attribution. GitHub does this though a website and adds features like issue tracking, collaborative (discussion based) code review, and team management. Anyone can download the code, work on an enhancement or issue, submit updates, fix issues, and discuss project goals, timelines, and features. The basic site has been built and users can let the developers (that’s all of us) know what needs to be added, changed or removed, and implement it themselves. Readers are encouraged to check out the ‘Projects’ page [44] for ideas on additional features/enhancements that you could work on.

## Conclusion

This paper describes a new project to support open spectral research data on the web. Anyone can contribute to the content, to the code, to the concept, or to the management/vision. This paper also outlines the components needed to put together such a project and it can be used as a template to build other websites with different functionality and/or different types of chemical data.

The current version of the OSDB is just a starting point. There are many additional features one can envision for the site and it is a hope that the reader has ideas of their own and adds them. Open source code has become a mainstay in the computing world. With the tools, concepts and frameworks outlined in this paper, open source research data will hopefully become a mainstay of the scientific community.

## Abbreviations

API: Application Programming Interface
ASDF: ASCII Squeezed Difference Form
CSS: Cascading Style Sheet
GUI: graphical user interface
HTML: Hypertext Markup Language
LAMP: Linux, Apache, MySQL, and PHP
JCAMP-DX: Joint Committee on Atomic and Molecular Physical Data—Data Exchange
JSON: JavaScript Object Notation
JSON-LD: JavaScript Object Notation for Linked Data
LDR: Linked Data Record
MAMP: Mac, Apache, MySQL, and PHP
MVC: model–view–controller
NMR: nuclear magnetic resonance
OOP: object oriented programming
OSDB: Open Spectral Database
OWL: web ontology language
PHP: pre-hypertext processor
REST: representational state transfer
SDM: scientific data model
SMILES: simplified molecular-input line-entry system
SPARQL: SPARQL protocol and RDF query language
SQL: structured query language
URI: uniform resource identifier
WAMP: Windows, Apache, MySQL, and PHP
XML: extensible markup language
